# Altered neutrophil immunophenotypes in childhood B-cell precursor acute lymphoblastic leukemia

**DOI:** 10.18632/oncotarget.8369

**Published:** 2016-03-25

**Authors:** Elen Oliveira, Thiago S. Bacelar, Juana Ciudad, Maria Cecília M. Ribeiro, Daniela R.N. Garcia, Lukasz Sedek, Simone F. Maia, Daniel B. Aranha, Indyara C. Machado, Arissa Ikeda, Bianca F. Baglioli, Nathalia Lopez-Duarte, Lisandra A. C. Teixeira, Tomasz Szczepanski, Maria Luiza M. Silva, Marcelo G.P. Land, Alberto Orfao, Elaine S. Costa

**Affiliations:** ^1^ Clinical Medicine Postgraduate Program, College of Medicine, Rio de Janeiro Federal University (UFRJ), Rio de Janeiro, Brazil; ^2^ Cytometry Service, Institute of Pediatrics and Puericulture Martagão Gesteira (IPPMG), UFRJ, Rio de Janeiro, Brazil; ^3^ Departament of Medicine and Cytometry Service, Cancer Research Center (IBMCC, USAL-CSIC), Institute for Biomedical Research of Salamanca (IBSAL), University of Salamanca (USAL), Salamanca, Spain; ^4^ Cytogenetics Service, IPPMG-UFRJ and Polo Xerém-UFRJ, Rio de Janeiro, Brazil; ^5^ Cytogenetics Department, Bone Marrow Transplantation Unit and Oncology Post Graduation Program, National Cancer Institute (INCa), Rio de Janeiro, Brazil; ^6^ Department of Pediatric Hematology/Oncology, Medical University of Silesia, Zabrze, Poland; ^7^ Service of Pediatric Hematology, Federal Lagoa Hospital (HFL), Rio de Janeiro, Brazil; ^8^ Service of Pediatric Hematology, São José do Avaí Hospital (HSJA), Itaperuna, Rio de Janeiro, Brazil; ^9^ Service of Pediatric Hematology/Oncology, Servidores do Estado Federal Hospital (HSE), Rio de Janeiro, Brazil; ^10^ Service of Pediatric Hematology, Children's Cancer Hospital of Barretos, Barretos, São Paulo, Brazil

**Keywords:** B-cell precursor acute lymphoblastic leukemia, residual hematopoiesis, altered neutrophil immunophenotype, multiparameter flow cytometry, childhood

## Abstract

An increasing number of evidences suggest a genetic predisposition in acute lymphoblastic leukemia (ALL) that might favor the occurrence of the driver genetic alterations. Such genetic background might also translate into phenotypic alterations of residual hematopoietic cells. Whether such phenotypic alterations are present in bone marrow (BM) cells from childhood B-cell precursor (BCP)-ALL remains to be investigated. Here we analyzed the immunophenotypic profile of BM and peripheral blood (PB) maturing/matured neutrophils from 118 children with BCP-ALL and their relationship with the features of the disease. Our results showed altered neutrophil phenotypes in most (77%) BCP-ALL cases. The most frequently altered marker was CD10 (53%), followed by CD33 (34%), CD13 (15%), CD15/CD65 (10%) and CD123 (7%). Of note, patients with altered neutrophil phenotypes had younger age (*p* = 0.03) and lower percentages of BM maturing neutrophils (*p* = 0.004) together with greater BM lymphocyte (*p* = 0.04), and mature B-cell (*p* = 0.03) counts. No significant association was found between an altered neutrophil phenotype and other disease features. These findings point out the potential existence of an altered residual hematopoiesis in most childhood BCP-ALL cases.

## INTRODUCTION

B-cell precursor acute lymphoblastic leukemia (BCP-ALL) is a genetically and clinically heterogeneous malignancy characterized by the clonal expansion of tumor B-cell precursors [1]. In the past, several distinct genetic/molecular subtypes of BCP-ALL have been identified which show an age-associated distribution with prognostic impact [2, 3]. Chromosomal alterations from such BCP-ALL-associated genetic subgroups appear to be associated with the development of the disease and they frequently are a hallmark of the tumor. Despite this, evidences exist about the existence of a genetic background that might involve the whole hematopoiesis and favor the development of BCP-ALL, e.g. in subjects with Down syndrome (DS) and a 30 fold increased risk for BCP-ALL [4], in individuals with germline mutations of the *RUNX1, PAX5, ETV6, GATA1, HOXA11, ANKRD26, MPL, TP53* and *RMB8A* genes [5–8] and in children with *RUNX1* amplification associated to t(15;21)(q10;q10) and a 3 × 10^3^ fold increase in the frequency of BCP-ALL [9]. In addition, around 2% of children with BCP-ALL present with a transient (preleukemic) aplastic phase, 2 to 9 months before initial diagnosis [10, 11]. In the past decades, efforts devoted to the analysis of BCP-ALL tumor cells have concentrated in the characterization of the oncogenetic steps that occur within the BCP-ALL blast cell compartment [2, 12]; in contrast, fewer studies have investigated the features of residual hematopoietic cells at diagnosis.

In addition to the above referred genetic predisposition to childhood BCP-ALL, other recent studies also provide further evidence about the potential involvement of the residual hematopoiesis in BCP-ALL; thus, the presence of genetic alterations which are typical of leukemic blast cells (e.g. *MLL* and *BCR-ABL1* gene rearrangements) have been also detected in mesenchymal stem cells [13] and residual neutrophils [14]. Altogether, these findings suggest that bone marrow (BM) hematopoiesis could be altered in at least a fraction of childhood BCP-ALL patients. Thereby, detailed characterization of residual hematopoietic BM and peripheral blood (PB) cells may contribute to a better identification of such patients.

In the past decade, evidences have accumulated which indicate that altered myeloid and lymphoid cell phenotypes are a surrogate marker for an altered hematopoiesis [15, 16], particularly in the elderly [17, 18]. Thus, phenotypic alterations of residual hematopoietic BM cells, such as those involving maturing neutrophils, monocytic and/or erythroid cells, as well as CD34^+^ myeloid precursors, have been associated with clonal hematopoiesis in patients with myeloid malignancies including (adult) acute myeloid leukemia (AML), myelodysplastic syndromes (MDS) and myeloproliferative neoplasms (MPN) [15, 16, 19]. Similarly, immunophenotypic alterations have also been reported in systemic mastocytosis in association with multilineage *KIT* mutations [20, 21] and a greater risk of progression to aggressive disease e.g. AML and MDS. More recently, altered MDS-like phenotypes involving maturing and mature neutrophils, monocytic and/or erythroid precursors, have also been found in adult patients with mature lymphoid malignancies such as multiple myeloma and monoclonal gammopathy of undetermined significance (MGUS) cases; of note, these patients frequently showed also MDS-related genetic changes in the phenotypically altered BM myeloid cells and CD34^+^ precursors [16]. Whether such phenotypic alterations are also present in BM cells from BCP-ALL patients remains to be investigated.

Here we investigated the presence and the frequency of altered patterns of expression of neutrophil-associated proteins in BM- and PB-derived maturing/mature neutrophils from children with BCP-ALL studied at diagnosis, and their potential relationship with tumor cytogenetics and the clinical behavior of the disease.

## RESULTS

### Altered neutrophil immunopheno types in the bone marrow and peripheral blood of children with BCP-ALL

**Figure 1:**
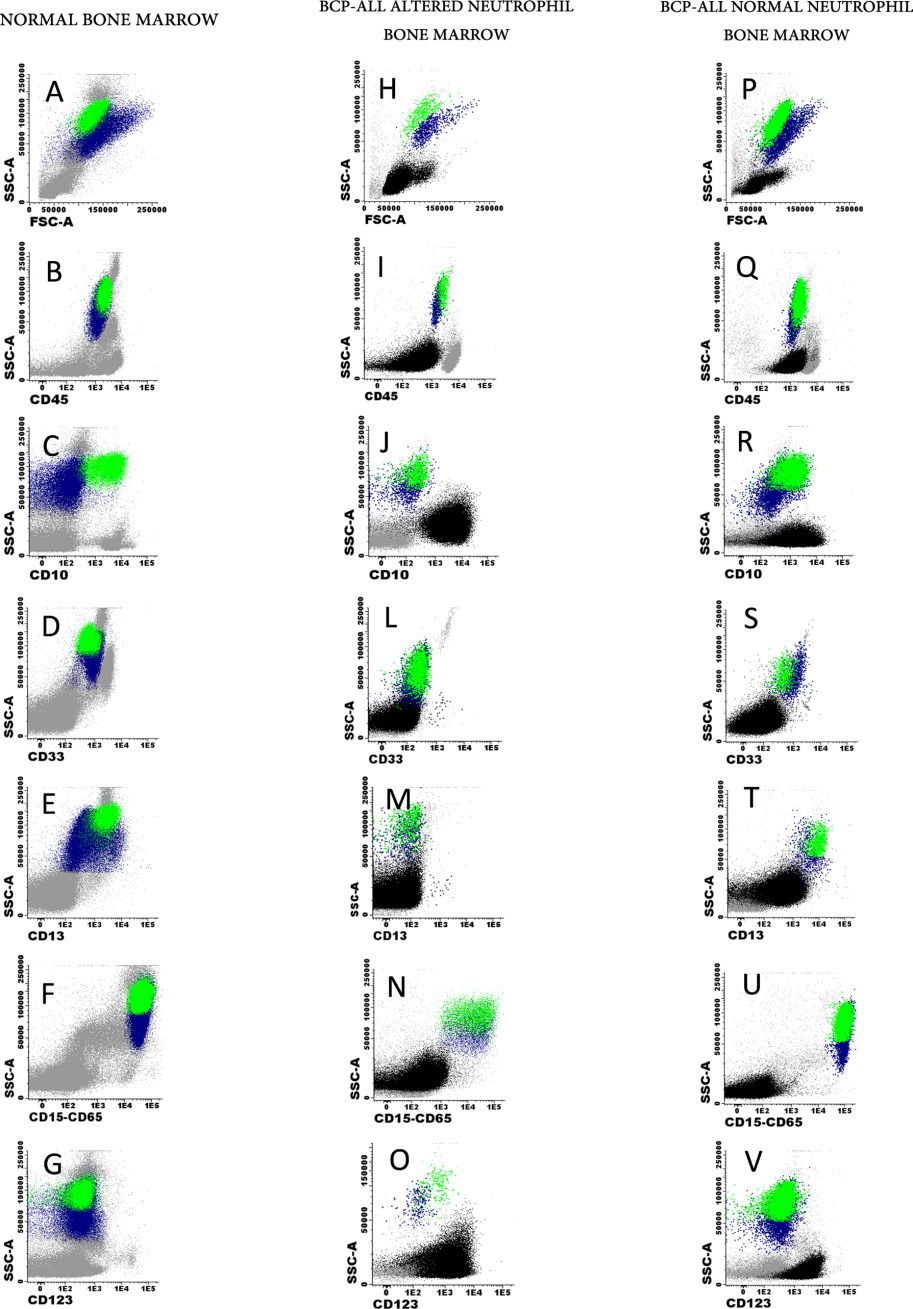
Illustrating examples of normal (A–G) and childhood BCP-ALL (H–V) BM mature/maturing neutrophil phenotypes for CD10 (C, J and R), CD33 (D, L and S), CD13 (E, M and T), CD15 plus CD65 (F, N and U) and CD123 (G, O and V) Mature neutrophils are depicted in green while neutrophil precursors are shown in blue. Blast cells and other BM cell compartmens are depicted as black and grey events, respectively.

**Figure 2:**
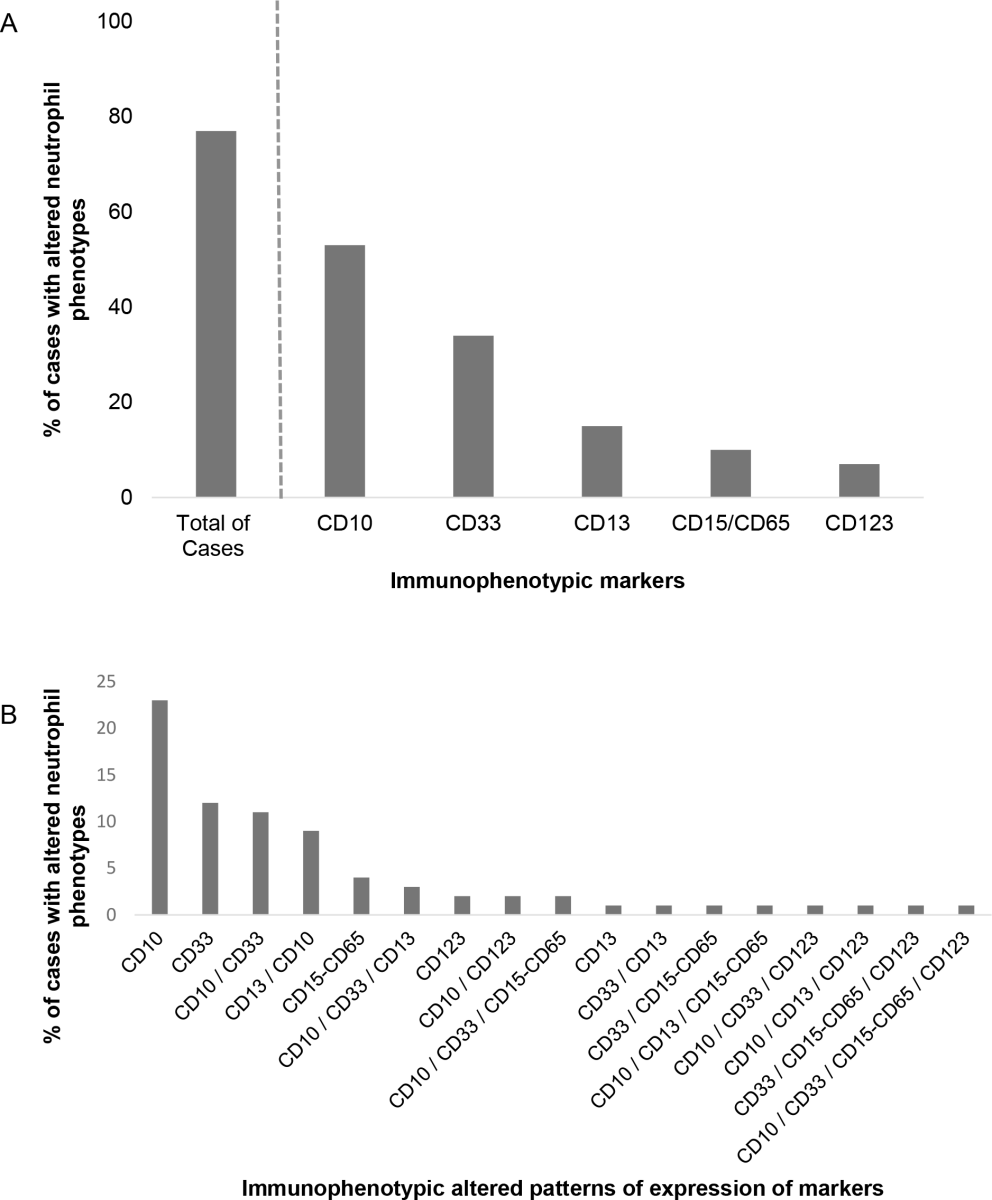
Frequency of BCP-ALL cases showing altered patterns of expression for individual (A) or multiple (B) markers on maturing/mature neutrophils

Altered maturing/mature neutrophil immunophenotypes were detected at diagnosis in the majority (85/110; 77%) of childhood BCP-ALL patients; in contrast, they were systematically absent in normal/reactive BM samples (0/12 cases; 0%) (*p* < 0.001), Figure 1. The most frequently altered marker was CD10, which was absent on BM and PB neutrophils of around half of the cases (62/118 patients; 53%) (*p* < 0.001). Absence of CD33 (36/107 patients; 34%) (*p* = 0.02), decreased expression of CD13 (17/111; 15%) (*p* = 0.14) heterogeneously low reactivity for CD15/CD65 (11/111; 10%) (*p* = 0.25) and overexpression of CD123 (8/109; 7%) (*p* = 0.33) (Figure 2A) were also found at variable frequencies. In more than one third of the patients (42/101; 42%) with altered neutrophil phenotypes these involved one marker; whereas two, three and four altered markers were found in 24/101 (24%), 9/101 (9%) and 1/101 (1%) BCP-ALL patients, respectively (Figure 2B). The most frequent combination of altered markers (11/101 cases; 11%) involved the absence of both CD10 and CD33 (Figure 2B).

Interestingly, no statistically significant differences were found (*p* > 0.05) between BM and PB samples, as regards the frequency of altered neutrophil phenotypes (79% in BM vs 58% in PB, *p* > 0.05), except for CD10 that was more frequently absent in BM than PB of childhood BCP-ALL (57% vs. 15%, *p* = 0.004). Of note, altered expression of CD10 on neutrophils did not correlate with the pattern of expression of CD10 on blast cells (*p* > 0.05).

In only 30% of cases (35/118) sufficient events (> 20) were measured that would allow analysis of the immuno phenotypic profile of CD34^+^/MPO^+^ neutrophil precursors. In all 35 cases, normal CD117 and CD13 expression patterns in the absence of CD7 were observed on CD34^+^/MPO^+^ precursors. In contrast, CD33 was expressed at abnormally low levels (i.e. negative) in CD34^+^/MPO^+^ precursors from 26% (9/35) of patients. Cases with an altered CD34^+^/MPO^+^/CD33^−/lo^ phenotype systematically showed also an altered maturing/mature neutrophil phenotypes (100% (9/9) vs. 65% (17/26), *p* = 0.03). Thereby, they more frequently displayed abnormal expression of both CD33–66% (6/9) vs 13% (3/23), *p* = 0.01 and CD15/CD65–100% (4/4) vs 13% (5/30), *p* = 0.003 - on maturing/mature neutrophils. Furthermore, the proportion of cases which had an altered phenotype on CD34^+^/MPO^+^ cells progressively increased in parallel to the number of aberrant markers observed in maturing/mature neutrophils, from 3/14 cases (21%) to 4/8 patients (50%) and 2/2 cases (100%) for BCP-ALL patients presenting with 1, 2 and ≥ 3 aberrant markers on mature/maturing neutrophils (*p* = 0.02).

After therapy, most patients showed (normal) recovery of CD10 expression on mature neutrophils. Thus, decreased or negative CD10 expression was observed in PBof 8/26 cases (31%) at day8 of prednisone therapy, and in the BM of 25/81 (30%), 12/96 (12%) and 6/87 (7%) of patients studied at day 15, 33 and 78 of therapy, respectively. Expression of other markers such as CD33, CD15/CD65, CD13 and CD123, on PB or BM neutrophils was not evaluated during follow up.

### Relationship between the presence of altered neutrophil phenotypes and the distribution of residual non-blast cell BM populations in childhood BCP-ALL

Children with BCP-ALL and altered neutrophil phenotypes showed a lower percentage of maturing neutrophils (19% vs. 28%, *p* = 0.004) and a greater proportion of lymphocytes (51% vs. 37%, *p* = 0.04), including mature B cells (8.8% vs. 7.1%, *p* = 0.03), than cases with normal neutrophil phenotypes (Table 1). In addition, the former cases also showed a significantly higher proportion of CD34^+^ myeloid precursors within the CD34^+^ (HPC) BM compartment (0.6% vs. 0.3%, *p* = 0.03) (Table 1). No statistically significant differences (*p* > 0.05) were found between both patient groups as regards the distribution of nucleated red blood cells (NRBC), monocytes, eosinophils, basophils, T lymphocytes and NK cells (Table 1).

**Table 1:** Distribution of non-blast cell subsets in the BM of children with BCP-ALL at diagnosis according to the presence vs. absence of altered immunophenotypes on residual maturing and mature BM neutrophils

Cell subsets	BCP-ALL with normal neutrophil phenotypes	BCP-ALL with altered neutrophil phenotypes	*p-*value
% of maturing neutrophils	28 (5–55)	19 (2–64)	*p* = 0.004
% of monocytes	0.92 (0.15–6.7)	1.04 (< 0.1–15)	*ns*
% of eosinophils	0.73 (0.07–8.1)	0.81 (< 0.1–5.5)	*ns*
% of basophils	0.18 (< 0.1–1.6)	0.21 (< 0.1–6.5)	*ns*
% of NRBC	11.1 (1–32)	9.12 (0.3–67)	*ns*
% of lymphocytes	37 (20–84)	51 (8.8–92)	*p* = 0.04
% of T cells	29 (15–75)	35 (5–75)	*ns*
% of NK cells	2.2 (< 0.1–7.9)	2.6 (< 0.1–27)	*ns*
% of mature B cells	7.1 (< 0.1–22)	8.8 (< 0.1–39)	*p* = 0.03
% of CD34^+^ myeloid precursors	0.3 (< 0.1–1.3)	0.6 (< 0.1–3)	*p* = 0.03
% of CD34^+^/MPO^+^ neutrophil precursors from all CD34^+^ HPC	8 (< 0.1–100)	< 0.01 (< 0.1–100)	*ns*
% of CD34^+^/CD7^+^ precursors from all CD34^+^ HPC	< 0.01 (< 0.1 – 13)	< 0.01 (< 0.1–88)	*ns*

Results expressed as median (range). BCP-ALL, B-cell precursor acute lymphoblastic leukemia; BM, bone marrow; NRBC, nucleated red blood cells; HPC, hematopoietic progenitor cells.

### Relationship between the presence of phenotypically altered (mature/maturing) neutrophils and the clinical features of the disease

**Figure 3:**
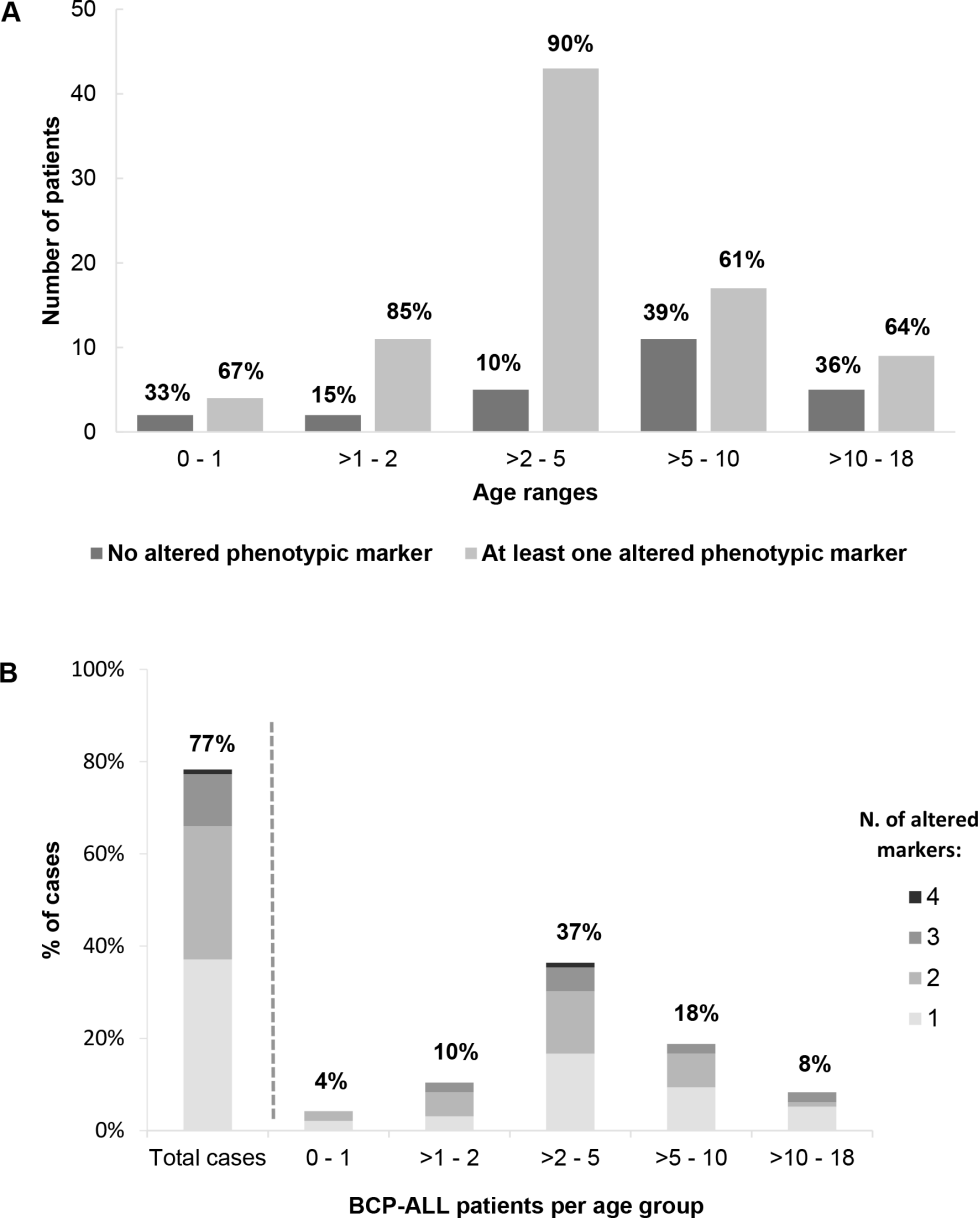
Frequency of childhood BCP-ALL cases with immunophenotypically altered maturing/mature neutrophils (**A**) The distribution of patients with immunophenotypically normal vs. altered neutrophils is represented by bars whose height represents the number of cases per age group. The relative distribution of patients with normal (dark gray bars) vs. at least one altered phenotypic marker (light gray bars) within each age group is shown in percentage numbers above the bar. In (**B**) bars indicate the relative distribution of cases (i.e. percentages) within each age group according the number of altered markers on neutrophils. The overall percentage of cases with an altered neutrophil immunophenotype is shown per age group, as numbers above the corresponding bar.

Interestingly, the frequency of cases with altered neutrophil phenotypes varied according to age; thus, BCP-ALL children with altered neutrophil phenotypes were younger than children who had normal phenotypes: median age at diagnosis of 4.0 vs. 6.5 years (*p* = 0.03) (Figure 3A–3B and Table 3). Thus, presence of at least one altered marker was detected in 62/82 (86%) BCP-ALL children younger than 6 years vs. 22/37 (60%) children older than 6 years (aged 6–16 years) (*p* = 0.002) (Figure 3A–3B). Overall, similar frequencies of altered neutrophil phenotypes were detected for the different markers investigated among BCP-ALL patients carrying distinct cytogenetic subtypes of the disease, except for a slightly lower frequency of cases showing (mature/maturing) neutrophils with a CD10^−^ and CD33^low^ phenotype among patients with *MLL* gene rearrangements - 27% vs 58% (*p* = 0.06), and 9% vs. 33% (*p* = 0.05) respectively - (Table 2). Regarding other features of the disease (Table 3), BCP-ALL children with altered patterns of expression of phenotypic markers on mature/maturing neutrophils showed similar PB cell counts, comparable frequencies of organomegalies, and overlapping rates of response to therapy, to those observed for cases who had normal neutrophil phenotypes.

**Table 2:** Distribution of aberrant patterns of antigen expression on bone marrow maturing and mature neutrophils from pediatric BCP-ALL patients studied at diagnosis: relationship with the genetic subtype of the disease

*Immunophenotypic marker*	*MLL rearrangement (n = 11)*	*Hyperdiploidy (n = 34)*	*t (12;21) (n = 21)*	*t (1;19) (n = 5)*	*Rare (n = 8)*	*Normal karyotype (n = 28)*	Total (n = 107)*	p-Value
**CD10**	**27%** (3/11§)	**53%** (18/34)	**67%** (14/21)	**80%** (4/5)	**63%** (5/8)	**50%** (14/28)	**58%** (62/107)	***ns***
**CD33**	**9.1%** (1/11§)	**31%** (9/29)	**45%** (9/20)	**20%** (1/5)	**25%** (2/8)	**42%** (10/24)	**33%** (32/97)	***ns***
**CD13**	**18%** (2/11)	**19%** (6/31)	**15%** (3/20)	**20%** (1/5)	**38%** (3/8)	**7%** (2/27)	**17%** (17/102)	***ns***
**CD123**	**9.1%** (1/11)	**6%** (2/32)	**17%** (3/18)	**20%** (1/5)	**25%** (2/8)	**7%** (2/28)	**8.8%** (10/114)	***ns***
**CD15/CD65**	**18%** (2/11)	**9%** (3/32)	**16%** (3/19)	**20%** (1/5)	**13%** (1/8)	**4%** (1/26)	**11%** (11/101)	***ns***
**≥ 1 altered phenotypic marker**	**73%** (8/11)	**77%** (24/31)	**85%** (17/20)	**100%** (5/5)	**75%** (6/8)	**76%** (19/25)	**79%** (79/100)	***ns***

The denominator in each fraction is the total number of patients with the genetic alteration evaluated for each specific marker.

* The total of patients was 107 because there were 11 cases with missing data concerning genetic alterations.

§ p-value < 0.05, vs. patients without MLL gene rearrangements.

**Table 3:** Relationship between the number of immunophenotypically altered markers on neutrophils and the clinical features of the disease

*Clinical features*	*Normal neutrophil phenotype*		*Altered neutrophil phenotype*	*P*-value
**Age (years)**	**6.5** (0.4–15)		**4.0** (0.3–16)	***0.03***
**Gender (male : female)**	**1.3 : 1**		**1 : 1.1**	***ns***
**Splenomegaly**	**62%**		**71%**	***ns***
**WBC count (× 10^9^/L)**	**4.9** (1.4–170)		**12.8** (1.2–450)	***ns***
**Neutrophil count (× 10^9^/L)**	**1.0** (0.2–12)		**0.8** (0.0–15)	***ns***
**% of patients with severe neutropenia (≤ 0.2 × 10^9^/L)**	0%		**11%**	***ns***
**Hemoglobin (g/dL)**	**8.3** (3.9–11.7)		**8.1** (4.2–13.5)	***ns***
**% of patients with severe anemia (≤ 6 g/dL)**	**13**		**19**	***ns***
**Platelet count (× 10^9^/L)**	**54** (12–275)		**40** (2–403)	
**% of patients with severe thrombocytopenia (≤ 30 × 10^9^/L)**	**27**		**40**	***ns***
**% of patients with ≥ 1 severe cytopenia**	**32**		**47**	***ns***
**Poor response to corticosteroids**	**11%**		**16%**	***ns***
**N. of blast cells in PB at day + 8 after therapy ( × 10^9^/L)**	**78** (0–1, 175)		**200** (0–202, 000)	***ns***
**BFM risk stratification**	**SR**	**21%**	**25%**	***ns***
	**MR**	**47%**	**44%**	
	**HR**	**32%**	**31%**	
**% of MRD-positive at day + 15 after therapy**	**68%**		**74%**	***ns***
**% of MRD-positive at day + 33 after therapy**	**41%**		**33%**	***ns***
**% of MRD-positive at day + 78 after therapy**	**16%**		**14%**	***ns***
**% 3-year event free survival**	**85%**		**84%**	***ns***
**% 3-year overall survival**	**82%**		**85%**	***ns***

## DISCUSSION

By definition, childhood BCP-ALL is a relatively heterogeneous group of *de novo* acute leukemias, which is characterized by an abnormal expansion of genetically altered B-cell precursors blocked at relatively early stages of B-lymphoid maturation [3]. Despite this, accumulating evidences suggest that a genetic predisposition might exist which favors the development of childhood BCP-ALL [4–9, 11]. Such genetic predisposition might translate into uniquely altered phenotypes of maturing residual hematopoietic cells that can mimic those observed in MDS, MPN and clonal hematopoiesis of indeterminate potential (CHIP) [22–25]. Here we investigated for the first time the presence of altered neutrophil phenotypes in a relatively large cohort of childhood BCP-ALL. Interestingly, three quarters of all childhood BCP-ALL cases studied showed altered phenotypes on BM and/or PB maturing/mature neutrophils. Abnormally low-to-negative expression levels of CD10, CD33, CD13 and CD15/CD65, together with overexpression of CD123, were recurrently detected at decreasing frequencies. These findings are in line with previous observations pointing out the existence of an altered neutrophil function in these patients at diagnosis, including decreased chemotaxis, phagocytosis and oxidative burst [26–28].

Of note, the presence of altered neutrophil phenotypes was associated with lower percentages of maturing neutrophils and higher lymphocyte counts in the BM, as well as with a younger age at diagnosis. In contrast, no significant associations were found between the presence of altered neutrophil phenotypes and PB cell counts, tumor cytogenetics and other clinical features of the disease, except for a lower frequency of altered CD10 and CD33 expression in patients with *MLL* gene rearrangement. This later association could not be due to technical artifacts such as antibody consumption due to high numbers of CD10^hi^ blast cells, among cases who show no *MLL* rearrangements (vs. *MLL* cases), since CD10 levels on blast cells did not show an association with CD10 expression levels on residual neutrophils. In addition, usage of different antibody clones in a subset of altered cases (*n* = 3) showed similar results, which would rule out also a reagent specific bias.

In recent years, several groups have independently reported the presence of clonal hematopoiesis in adults diagnosed with both *de novo* AML and mature lymphoid neoplasias (e.g. MM and MGUS), as well as in otherwise healthy adults [17, 22, 29–31]; of note, the frequency of leukemia and cancer cases increased with age among later group [23–26]. Based on these observations, it has been hypothesized that the presence of clonal hematopoiesis in the absence of clear cut criteria for a hematological disorder might precede (or even favor) the development of both myeloid and lymphoid malignancies, at least in a fraction of cases. Despite this, no study has been reported so far in which the presence of cytogenetic and/or phenotypic markers associated with clonal hematopoiesis have been systematically investigated in residual BM myeloid cells from children with BCP-ALL.

Here we report a high frequency of altered (e.g. MPN/MDS-like) phenotypes on BM and PB neutrophils of children with BCP-ALL; these findings would support the existence of an altered hematopoiesis also in childhood BCP-ALL. In line with this, patients with altered neutrophil phenotypes also showed reduced BM neutrophil counts vs. cases displaying normal neutrophil phenotypes, independently of the genetic subtype of BCP-ALL. These findings suggest a more pronounced impairment of neutrophil production among the former group of patients. Despite this, no statistically significant differences were observed between BCP-ALL children with normal vs. altered neutrophil phenotypes, as regards PB neutrophil counts. Such lack of association could be due to the fact that PB neutrophil counts also depend on factors other than a clonal hematopoiesis, such as the degree and the pattern of BM involvement by blast cells, the BM microenvironment and/or the local production of growth factors and cytokines, in addition to the specific underlying alteration of residual (potentially clonal) hematopoiesis. In fact, disruption of the normal HPC BM niches caused by leukemic cell growth has recurrently been reported [32, 33] and deserves further (ongoing) investigations.

Independently of whether or not the presence of altered neutrophil phenotypes might be due to an underlying genetically altered (clonal) hematopoiesis, it might be speculated that this would provide an environment that might favor an earlier onset of the disease. Thus, children with BCP-ALL and altered neutrophil phenotypes also showed a significantly lower age at disease onset. In contrast, no significant differences were found in the clinical behavior of the disease and patient outcome, depending on the presence vs. absence of altered neutrophil phenotypes. Altogether, these observations suggest that the course of BCP-ALL more likely depends on features of the disease related to the tumor cells, tumor burden and response to therapy, than to the presence of an underlying altered hematopoiesis, at least in the short-to-medium term, which is also supported by the progressive recovery of CD10 expression on neutrophils from treated BCP-ALL cases here reported. In turn, such lack of clinical correlation could also be due to the fact that we only investigated the presence of altered phenotypes on neutrophil lineage cells, but not on other relevant hematopoietic cell compartments (e.g. maturing monocytes and NRBC) which might also be abnormal, even in cases that showed normal neutrophil immunophenotypic patterns. Interestingly, our results showed a close association between the presence of altered phenotypes on CD34^+/^MPO^+^ neutrophil precursors and a greater number of aberrant phenotypes on maturing/mature neutrophils. These results might suggest that alteration of a single marker such as CD10 might not be as relevant as the coexistence of multiple phenotypic alterations involving also precursor cells, in line with what has been previously reported for MDS patients [24]. Further studies, in which the presence of altered phenotypes is investigated in BM/PB cell compartments other than neutrophils, are required to confirm this hypothesis.

Despite all the above, it might be speculated that the herein reported altered phenotypes might not be specific for an underlying clonal hematopoiesis, since they may also be found in other reactive non-clonal conditions. In this regard, absent-to-low expression of CD10 has been reported for mature neutrophils from septic shock patients [34] in association with a greater chemotactic response to activated complement (vs. CD10^+^ neutrophils) [35]. Similarly, decreased CD13 levels have been reported on neutrophils from HIV^+^ patients in parallel to the decrease in PB CD4^+^ T cell counts [36]; in these patients, greater CD13 levels have an inhibitory effect on TNFα induced apoptosis [37]. In fact, CD10 and CD13 can be downregulated by cytokines, and growth factors such as TNFα, and GM-CSF [28]. Thereby, the time span from disease onset to diagnosis could contribute to the decreased expression of CD10 and/or CD13, due to more prolonged time of exposure to an inflammatory microenvironment. Regarding altered expression of other markers here investigated in reactive conditions, low expression of CD15 on mature neutrophils has also been reported among individuals with delayed asthmatic response, suggesting the involvement of a cell-mediated hypersensitivity mechanism in bronchial asthma [38]. Concerning CD33, no clear association has been reported between decreased levels of expression of this molecule and other non-neoplastic conditions, except for a rare single nucleotide polymorphism that prevents binding of specific anti-CD33 antibody clones to CD33 [39]. In contrast, greater levels of CD123 on neutrophil lineage cells have been recurrently reported in association with clonal hematopoietic disorders [40, 41]. Altogether, these findings support the notion that, despite some of the herein described phenotypic alterations might occur in rare disease conditions or small groups of individuals, these could not explain the rather high frequency of phenotypically altered neutrophils observed among our BCP-ALL patients.

In summary, here we describe for the first time, the presence of altered phenotypes on neutrophils from children with BCP-ALL, in association with an earlier onset of the disease. Such findings support the existence of an altered residual hematopoiesis in most childhood BCP-ALL cases. Whether such altered phenotypes also reflect an underlying clonal hematopoiesis in a substantial fraction of childhood BCP-ALL patients or they are a consequence of massive leukemic infiltration of the BM microenvironment, deserves further investigations.

## MATERIALS AND METHODS

### Patients and samples

Overall, 105 BM and 13 PB samples from 118 children diagnosed with BCP-ALL by the WHO criteria (59 males and 59 females; mean age: 6 ± 4 years, ranging from 0.3 to 16 years), were studied. No paired BM and PB samples were analyzed. In parallel, normal and reactive BM samples from 12 children (6 males and 6 females; mean age of 6 ± 6 years, ranging from 0 to 15 years), were also studied as controls. Samples were obtained at three different institutions – Institute of Pediatrics and Puericulture Martagão Gesteira, Rio de Janeiro Federal University (IPPMG/UFRJ), Rio de Janeiro (Brazil); University Hospital of Salamanca, University of Salamanca, Salamanca (Spain), and; Medical University of Silesia, Zabrze (Poland) – after informed consent was given according to the Helsinki Declaration protocol. The study was approved by the local Ethics Committees. Patients were treated with the IC-ALL-BFM 2002 (32% - 38/118), IC-ALL-BFM 2009 (34% - 40/118), INTERFANT 2006 (5% - 6/118) and SEHOP-PETHEMA LAL 2013 (28% - 33/118) protocols. Of note, a high number of infants were studied since IPPMG/UFRJ is a reference center for infant ALL.

### Multiparameter flow cytometry immunophenotypic studies

Individual BM and PB samples were obtained at diagnosis prior to the administration of any therapy, and were stained with the EuroFlow 8-colour – fluorescein isothiocyanate (FITC)/phycoerythrin (PE)/PE-cyanin7(PECy7)/peridinin chlorophyll protein-Cy5.5 (PerCP-Cy5.5)/allophycocyanin (APC)/APC-hilite7 (APC- H7)/pacific blue (PacB)/pacific orange (PacO) – acute leukemia orientation tube (ALOT) plus the BCP-ALL panel [42]: *i)* cytoplasmic (Cy) MPO/_Cy_CD79a/CD19/CD34/CD7/surface membrane (Sm) CD3/_Cy_CD3/CD45; *ii)* CD58/CD66c/CD19/CD34/CD10/CD38/CD20/CD45; *iii)*_Cy_IgM/CD33/CD19/CD34/CD117-_Sm_IgM/_Sm_Igλ/ _Sm_Igκ/CD45; *iv)* nuclear (Nu) TdT/CD13/CD19/CD34/CD22/CD24/CD9/CD45; *v)* CD15-CD65/NG2/CD19/CD34/CD123/CD81/CD21/CD45. Staining of BM and PB cells was performed as previously described in detail, according to the standard operating procedures proposed by the EuroFlow Consortium [43]. Immediately after staining, cells were acquired in a FACSCanto II flow cytometer – Beckton/Dickinson Biosciences, San José, CA (BD), using the FACSDiVa software (BD). For data analysis, the INFINICYT™ software (Cytognos SL, Salamanca, Spain) was used. Mature neutrophils were identified as SSC^hi^, FSC^int^ and CD45^hi^ cells; maturing neutrophil precursors were defined as SSC^int-to-hi^, FSC^int-to-hi^ and CD45^lo^ cells (Figure 1), both in normal (Figure 1A–1G) and BCP-ALL samples (Figure 1H–1O). Altered neutrophil phenotypes were defined by the absence or abnormally low (< 2 SD of normal BM neutrophils) expression levels of CD10, CD33 and CD15/CD65 and/or by aberrantly high expression levels of CD123 [44].

### Cytogenetic and fluorescence *in situ* hybridization FISH studies

Cytogenetic studies were performed on short-term (2–72 h) cultured BM or PB cells collected at diagnosis and processed by standard procedures [45]. Conventional cytogenetic analysis was performed by GTG or QFQ banding using the Band View 5.5 software (Applied Spectral Imaging, Vista, CA, USA) or the IKAROS 5.4 software (MetaSystems, Altlusshein, Germany). Karyotypes were reported following International System for Human Cytogenetic Nomenclature (ISCN 2013).

FISH analyses were performed on methanol/acetic 3/1 (v/v) fixed cells. Slides were prepared, hybridized and analyzed using commercially available probes following the manufacturer's recommendations. Locus-specific DNA probes (LSI Abbott VYSIS, Des Plaines, IL, USA) were used to detect *ETV6-RUNX1* (Dual-color, dual fusion), *MLL*(Dual-color, break apart) and *BCR-ABL* (Dual-color, dual fusion) gene rearrangements. Fluorescence signals were detected using a BX-51 epifluorescence microscope (Olympus, Miami, FL, USA) equipped with the ISS 5.4 software (MetaSystems) and the Fish View 5.5 software (Applied Spectral Imaging). The number of mitosis and interphase nuclei investigated by classical cytogenetics and FISH was of 20 metaphases (whenever available) and > 200 interphase nuclei, respectively. Multicolor banding FISH was performed in cases with complex karyotypes [46] and fluorescence signals were detected using an AXIO- Image D2 epifluorescence microscope (ZEISS, Thornwood, NY, USA) equipped with the MetaArquive 5.2 software (MetaSystems).

### Statistical methods

In order to establish the statistical significance of differences observed between groups, the Kruskall-Wallis and either the Mann-Whitney *U* tests (for continuous variables) or the Chi square test (for categorical variables) were used (SPSS software package, version 18.0, SPSS Inc., Chicago, IL, USA). *P*-values < 0.05 were considered to be associated with statistical significance.
